# Bright Light Therapy for Major Depressive Disorder in Adolescent Outpatients: A Preliminary Study

**DOI:** 10.3390/clockssleep6010005

**Published:** 2024-01-30

**Authors:** Rachel Ballard, John T. Parkhurst, Lisa K. Gadek, Kelsey M. Julian, Amy Yang, Lauren N. Pasetes, Namni Goel, Dorothy K. Sit

**Affiliations:** 1 Ann & Robert H. Lurie Children’s Hospital of Chicago, 225 E. Chicago Ave., Box 10, Chicago, IL 60611, USA; rballard@luriechildrens.org (R.B.); jtparkhurst@luriechildrens.org (J.T.P.); kjulian@luriechildrens.org (K.M.J.); 2 Lake Forest Pediatrics, Lake Bluff, IL 60044, USA; lgadek@lakeforestpediatrics.com; 3 Asher Center for the Study and Treatment of Depressive Disorders, Department of Psychiatry and Behavioral Sciences, Northwestern University Feinberg School of Medicine, 676 N. St. Clair St., Suite 1000, Chicago, IL 60611, USA; yangy.ustc@gmail.com; 4 Biological Rhythms Research Laboratory, Department of Psychiatry and Behavioral Sciences, Rush University Medical Center, 1645 W. Jackson Blvd., Suite 425, Chicago, IL 60612, USA; lauren_n_pasetes@rush.edu (L.N.P.); namni_goel@rush.edu (N.G.)

**Keywords:** adolescent, depression, bright light therapy, placebo comparator, sleep, primary care

## Abstract

Background: Bright light therapy (BLT) has not been well-studied in adolescents with major depressive disorder, particularly in outpatient settings. Methods: We conducted an 8-week clinical trial of BLT in adolescents recruited from a primary care practice with moderate to severe major depression. Acceptability and feasibility were defined by daily use of the light box and integration into daily routines. To assess treatment effects, we utilized the Short Mood and Feelings Questionnaire (SMFQ) and actigraphic sleep variables. Results: Of the nine enrolled adolescents, the rate of daily use of the light therapy box was 100% at week 2, 78% at week 4 (n = 7), and 67% at weeks 6 and 8 (n = 6). Participants were better able to integrate midday BLT compared to morning BLT into their day-to-day routines. Mean depression scores improved during the 2-week placebo lead-in (dim red light—DRL) and continued to show significant improvement through 6 weeks of BLT. Sleep efficiency increased significantly (*p* = 0.046), and sleep onset latency showed a trend toward a significant decrease (*p* = 0.075) in the BLT phase compared to the DRL phase. Conclusion: Bright light treatment that was self-administered at home was feasible, acceptable, and effective for adolescent outpatients with depression. Findings support the development of larger, well-powered, controlled clinical trials of BLT in coordination with primary care.

## 1. Introduction

Major depressive disorder (MDD) in adolescents is a major public health concern, with a 12-month prevalence rate of 7.5% in the United States [1]. Adolescent major depression is a significant risk factor for suicide, social and educational impairment, and significant societal costs [2]. Psychotherapies and antidepressant medications, primarily selective serotonin reuptake inhibitors (SSRIs), are effective in the treatment of adolescent depression. Combination therapy (psychotherapy and medication management) is considered the optimal treatment approach [3]. Barriers to this treatment include a lack of available psychotherapists and psychiatric clinicians, hesitancy in prescribing SSRIs among primary care clinicians, and low acceptance of antidepressants by patients and families. Moreover, up to 40% of adolescents with major depression may not improve with adequate combined treatment [3,4]. 

Bright light therapy (BLT) is an affordable, non-pharmacologic intervention for MDD that can be delivered conveniently in home or school settings and may be an acceptable option for outpatient adolescents with depression. BLT is effective in the treatment of seasonal affective disorder [5,6,7,8], non-seasonal depression [9,10,11], perinatal depression [12], and bipolar depression in adults [13,14]. Treatment with BLT offers a similar level of effectiveness as antidepressant medications when implemented as a stand-alone intervention or as an augmented strategy in combination with antidepressants [15]. Daily exposure to bright, white light (at intensities of 7000 lux to 10,000 lux) provides a robust and superior response over plausible placebos for adults with major mood disorders [10,16]. Despite the large evidence base in adults, BLT in children and adolescents is less well-studied [17].

Adolescents are susceptible to environmental cues that can alter circadian rhythms and also precipitate sleep disturbance and mood dysregulation [18]. Seasonal variation in mood and behaviors [19], sleep phase delay [20], and atypical features of depression such as symptoms of increased appetite, weight gain, hypersomnia, fatigue, and rejection sensitivity [21], all of which characterize MDD in youth, are strong predictors of responsivity to BLT in adults [22]. Given the clinical characteristics of depressed youth, and the complexity and limitations of antidepressant drugs in adolescents, BLT presents a promising non-drug, somatic option, which may optimally target the mood symptoms most frequent in adolescent depression.

Well-powered studies to support BLT as a treatment for depressive disorders, especially in outpatient adolescents, are lacking (see scoping review by Ballard et al. [17]). Early trials of outpatient BLT mainly investigated children and adolescents with seasonal affective disorder (SAD). Only four randomized controlled trials and two crossover studies of BLT have been conducted on adolescents with non-seasonal depression; the majority (except for one) examined inpatients. The inpatient trials used 10,000 lux units (light boxes or, in one study, light glasses) delivered for 30–60 min in the morning in addition to other inpatient treatment modalities including psychotherapies and medication. The trials that included a placebo control for BLT used dim light (150–200 lux) [17]. Two of these trials demonstrated significant improvement among adolescents with complex depression who had anorexia nervosa [23] or cancer-related fatigue [24] and represented outcomes among important but less-generalized populations.

The effects of BLT on adolescents in inpatient treatment may not extrapolate to treatment in the outpatient setting. Inpatients may be more ill and have comorbidities, but their adherence to treatment can be facilitated and closely monitored compared to that of outpatients. The feasibility or acceptability of the use of BLT for any duration has not been fully established in adolescent outpatients with contemporary units. The dose-finding studies [25] carried out for adults with bipolar depression have not been conducted among adolescent patients. The minimum daily light exposure duration and the timing to achieve symptom response have yet to be determined in this group.

Given this limited knowledge, we conducted a preliminary and hypothesis-generating study in an outpatient clinic setting to investigate three main study aims: 1. to characterize and define facilitators/barriers to treatment with BLT in adolescents; 2. to evaluate the acceptability and feasibility of outpatient BLT in a dose titration protocol; and 3. to establish an effective, safe, and tolerable light dose.

## 2. Results

### 2.1. Clinical Characteristics

Fourteen participants completed the initial assessment. One participant was deemed ineligible due to concerns about manic symptoms and two were deemed ineligible due to subthreshold depression. Of the remaining 11 participants, one interested candidate declined enrollment due to time constraints and one preferred to pursue antidepressant treatment; nine participants enrolled in the BLT trial. Of these nine participants, the mean age was 14.9 years, SD 2.2 years. Six were female and three were male. Race and ethnicity were represented as follows: five participants were White, non-Hispanic; one participant was Black, non-Hispanic; one participant was mixed White/Asian, non-Hispanic; one participant was mixed White/Puerto Rican, Hispanic; and one participant indicated “preferred to not answer” regarding their race. Of the enrolled youth, three (33%: #3, #4, and #9) showed seasonal traits and winter-related mood worsening (global seasonality score of 11 or more). Five of the nine participants (56%: #2, #4, #5, #10, and #14) described an evening preference (MEQ score less than or equal to 41) compared to four participants (44%: #3, #7, #8, and #9) who described an intermediate preference (MEQ score 42–48). *Comorbid diagnoses*. Five of the enrolled participants had one or more secondary diagnoses on the MINI-KID that included social anxiety disorder (two participants), eating disorder (two participants, one with restrictive eating and one with binge eating), attention deficit hyperactivity disorder (one participant), Tourette syndrome (one participant), and substance use (one participant).

### 2.2. Acceptability and Feasibility

Of the nine participants, six completed the 8-week study protocol; they attended study visits, used the study light boxes as directed, and filled out the study assessments. Treatment acceptance was defined by daily use of the light box. From week 0 to week 2, all participants (nine out of nine) used the light box as prescribed (30 min/d DRL). By week 4, seven out of the nine participants used the light box daily (15 min/d BLT) and two participants (#7 and #9) stopped light therapy (one participant started a summer job mowing lawns and had no time to use the light box, and the other went away to visit cousins and did not take the light box with them). Through week 6 to week 8, six out of the nine participants continued to use the light box (15–45 min/d BLT) and three participants (#7, #8, and #9) completely stopped light therapy. In summary, the participation rate defined as daily use of the light therapy box was 100% at week 2, 78% at week 4, and 67% at weeks 6 and 8.

In the initial recommendation, participants were advised to use the light box within 30 min of waking, according to the standard protocol [26]. Not surprisingly, finding time in the morning to use the box before school was a challenge for several participants. The non-blinded team member worked individually with participants and their parents to establish the most feasible time of day to use the light box treatment. Seven participants initiated 30 min of DRL in the morning; because of time constraints (school bus pick up), two participants (#4 and #5) initiated DRL at midday. At week 2, two different participants (#2 and #3) requested midday BLT whereas the others continued morning BLT. At week 4, three participants received light therapy in the morning and the other three received it at midday. By week 6, one participant adhered to morning BLT and the others adhered to midday BLT. At week 8, all six participants requested midday light, but the application to their routines varied a little depending on individual needs. One participant utilized midday BLT primarily on weekends and morning BLT on weekdays before summer school; two used either midday BLT (~1500 h after school) or afternoon BLT (~1730 h after soccer practice) every day; and three selected a divided dosing strategy and received half of the total BLT dose in the morning and the other half at midday. The three non-completers only trialed morning BLT and declined alternate times for BLT. Of the six completers, two participants experienced a significant change in depression mood scores from 15 min/d of BLT and four experienced a significant mood change from 45 min/d of BLT.

### 2.3. Mood Ratings

**Figure 1:**
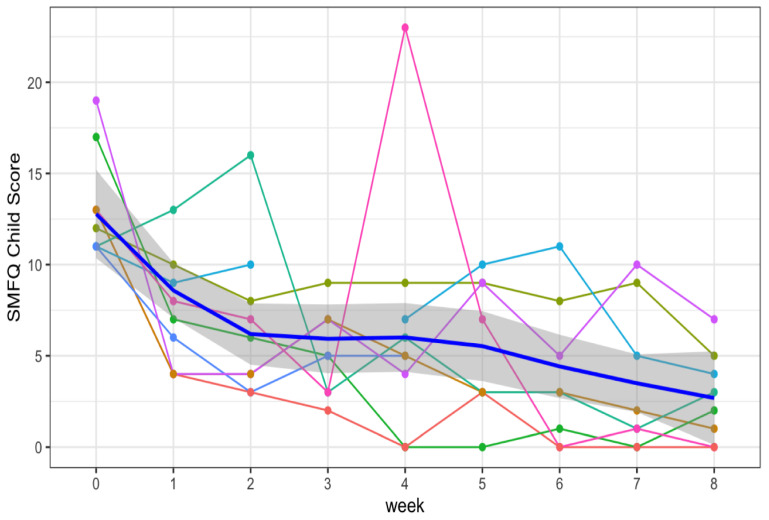
SMFQ child mood scores across study duration with a 95% confidence interval band (LOWESS curve is depicted in grey). In this graph, the SMFQ child scores showed the steepest improvement between baseline, week 0, and week 2 (median 13 vs. median 6). From week 2 to week 8, symptom score continued to improve but at a slower rate (median 6 vs. median 2.5). Each colored line represents an individual participant’s SMFQ scores across the study duration.

Individual SMFQ scores across study duration are shown in Figure 1. At baseline, participants endorsed a moderate level of depression (median SMFQ = 13, interquartile range (IQR) = 11–13) that was consistent with clinician ratings of moderate severity (median CGI-S = 4, IQR = 4–5). The parents recognized a mild level of depression severity (median parent SMFQ = 7, IQR = 7–9). The correlation between participant and parent SMFQs was Rho = 0.56 (using Spearman’s correlation coefficient). Comparisons of within-person change in SMFQ mood scores using non-parametric paired tests (using Wilcoxon signed-rank tests) showed significant changes with large effect sizes (Cohen’s d) [27] from exposures to DRL (weeks 0–2, median change = 7, V = 42, *p* = 0.042, d = 1.10) and DRL followed by BLT (weeks 0–8, median change = 10, V = 36, *p* = 0.042, d = 3.52), and a directional effect from exposure to BLT (weeks 2–8, median change = 4, V = 32, *p* = 0.058, d = 0.99; see Table 1). The within-person changes in parent SMFQ mood scores showed a directional effect from exposure to DRL followed by BLT (weeks 0–8, median change = 7, V = 28, *p* = 0.066; d = 1.35) but no significant effects from exposures to DRL or BLT (see Table 1). Clinician ratings indicated only directional effects of within-person CGI-S changes from 2 weeks of DRL (weeks 0–2, median CGI-S change = 2, V = 21, *p* = 0.067; d = 1.12), from DRL followed by BLT (weeks 0–8, median CGI change = 2, V = 28, *p* = 0.064; d = 2.00), and from BLT (weeks 2–8, median CGI = 1, V = 15, *p* = 0.067; d = 1.73). A clinically reliable change score [28] of 5.5 was calculated for the SMFQ using available data [29,30]. The percentage of youth reported meaningful SMFQ improvement was 67%, 37.5%, and 100% for the baseline to week 2 period, the week 2 to week 8 period, and the baseline to week 8 period, respectively. The relative change (change score/baseline score) was 65%, 24%, and 80% for the baseline to week 2 period, the week 2 to week 8 period, and the baseline to week 8 period, respectively.

Anxiety symptoms were reported by all participants (n = 9) at baseline, with a median child-reported total SCARED score of 34 (IQR = 32–38) and a median parent-reported total SCARED score of 11 (IQR = 4–21). At baseline, two of the parents’ measures and seven of the youth measures were elevated above the clinical cut off of 25 on the SCARED. Within-person changes in anxiety scores were not significant from exposures to DRL, BLT, or DRL followed by BLT. Neither youth nor parent reports showed clinically meaningful changes from baseline to 8 weeks (reliable change calculated as required to have a >21- or >14-point change in SCARED total score for youth and parent, respectively.)

### 2.4. Adverse Events

No participants withdrew from the study due to adverse events. There was no significant change in the SAFTEE score across study intervals. On the repeated SAFTEE (26-item modified), more than 50% of participants reported general aches, headaches, or eye irritation that ranged from “a little bit” to “moderate” in intensity, at baseline and throughout the study. Participants also reported physical symptoms of feeling agitated, dizzy, restless, or sleepy, or having stomach pains or a racing heart that improved across visits. Of the six participants who reported menstrual cycles, five had irregular periods, four experienced heavy bleeding, and two experienced mild spotting. There were no instances of treatment-emergent mania. The CMRS scores remained low (0–2) throughout the study. One study participant (#14) reported subtle hypomanic symptoms at week 4 (CMRS = 3) and week 6 (CMRS = 5) that completely resolved by week 8 (CMRS = 0). On the CSSRS safety measure, only one participant reported a lifetime history of suicidal behaviors (without hospitalization) at baseline. None of the participants indicated suicidal symptoms during the study.

### 2.5. Expectations

On the expectations question at baseline, seven youths expected major improvement and the other two participants (#4 and #5) expected minimal improvement. At the final visit, three participants reported that their symptoms had very much or much improved (participants #3, #9, and #14), four participants reported minimal or no improvement (#2, #5, #8 and #10), and two participants did not provide responses (#4 and #7).

### 2.6. Actigraphy Measures

Sleep efficiency (the percentage of time spent asleep out of the total sleep time) showed a statistically significant increase (z= −1.99, *p* = 0.046), and sleep onset latency (the time it takes to fall asleep) showed a trend toward a significant decrease (z= −1.78, *p* = 0.075) in the BLT phase compared to the DRL phase (see Table 2). There were no statistically significant differences in other actigraphy-estimated characteristics including sleep onset, sleep offset, sleep midpoint, sleep duration, wake after sleep onset, percentage of wake or sleep, or total sleep time between phases (all *p* > 0.05; see Table 2). There were also no significant differences in actigraphic total activity counts, or in white, red, green, or blue light exposure detected at the wrist between phases (all *p* > 0.05).

## 3. Discussion

Bright light treatment self-administered at home was safe and acceptable to adolescent participants with depression. Acceptability was facilitated by allowing the adolescent participants to specify a time of day in which they could integrate light therapy into their daily routines. The timing of light therapy was initially planned for early morning before school, but as the duration of light exposure increased, most participants found it more feasible to switch some or all their light therapy time to after-school hours. The overall adverse effects of light therapy were tolerable and similar to effects reported in adults [26] and children/adolescents with depression [17]. There were no adverse effects associated with morning, midday, or afternoon use. Specifically, there was no disturbance of sleep associated with light therapy administered at midday or in the afternoon. Bright light therapy was associated with decreased depressive symptoms from adolescent ratings and with improved sleep; clinician ratings showed a trend toward decreased depressive symptoms. Anxiety symptoms did not change significantly.

### 3.1. Recruitment

We recruited study participants from primary care pediatric practices, which allowed us to identify treatment-naïve individuals who entered the study with the endorsement of a trusted medical professional. Referring pediatricians expressed interest in being able to provide accessible, effective treatment options for their adolescent patients experiencing depression.

### 3.2. Feasibility and Acceptability in Adolescent Outpatients

Our study started with a question: will adolescents use a light therapy box for a prescribed duration at a prescribed time every day? Our stakeholder panel indicated that adolescents were skeptical about working light therapy into a daily schedule. In contrast to published studies on light therapy for non-seasonal adolescents conducted in inpatient units with clinical supervision, our participants were requested to incorporate light therapy into their daily routines of school and extracurricular activities. Parents were asked to support their teens and to report on symptoms, but the ultimate responsibility for engaging with the light therapy box remained with the adolescents. A key finding from this strategy is that mandated early morning light therapy may be neither feasible nor acceptable for many adolescent outpatients (at least during the school year), but that flexibly timed light therapy is feasible, acceptable, and well-tolerated.

In this study, the rates of participation were consistent with other trials of BLT in adolescents. Among controlled trials lasting up to two weeks, the dropout rates were 4% [31], none [32,33], or not provided [34]. In a trial lasting eight weeks, the light box was used on 57% of the days [24]; in a 6-week trial the dropout rate was not provided [23], and in a trial where BLT was prescribed three days per week for three weeks, the dropout rate was 7% [35].

### 3.3. Parent–Adolescent Correlation in Assessment of Adolescent Depression

The parents’ assessment of their adolescent children’s depressive symptoms was lower than the adolescents’ self-assessment. This is consistent with the literature in which the parent–child correspondence of youth depressive symptoms using Pearson’s correlation coefficients range from 0.25 to 0.45 [36].

### 3.4. Anxiety

Our participants presented with significant anxiety symptoms as well as depression, a common pattern in adolescents [37]. To date, there have been no studies of BLT in the treatment of child or adolescent anxiety disorders, with or without comorbid depression. Further study is needed to assess the role of anxiety in moderating the response of depression to BLT as well as any direct response of anxiety to BLT.

### 3.5. Placebo Response and Expectations

The large and significant treatment effects demonstrated by improved mood scores during DRL that further improved during treatment with 10,000-lux BLT are similar to earlier reports that showed positive responses to dim 300-lux yellow light in two cases [19], and to placebo dim white light (200 lux and 50 lux, respectively) followed by crossover to 2500-lux BLT [34,38]. The larger treatment effect shown with BLT preceded by DRL (weeks 0 to 8) compared to DRL (weeks 0 to 2) was a non-conclusive finding that may have been related to differences in the sequence of exposure (DRL followed by BLT versus only DRL) or to the length of exposure (8 weeks versus 2 weeks) or to some non-specific factor. A parallel placebo-controlled design with a power analysis to inform the sample size for recruitment is a preferable approach to demonstrate efficacy and treatment effect of an intervention compared to placebo [14] or of two equivalent interventions [39]. As systematically reviewed by Locher et al. [40], patient expectations of treatment, particularly their perceptions and belief in the credibility of the clinician and the treatment methods, spontaneous improvement in depression severity (less likely among our moderate-to-severely depressed participants), and active participation in the control condition (as required by our participants who agreed to daily use of the non-active unit during the lead in phase) are aspects of this study that may explain some of the positive treatment effects that we observed. Intriguingly, the high expectations for major improvement endorsed by participants at baseline, which diminished across the duration of the study, suggest that additional factors imparted the enduring clinical response to BLT that we detected.

### 3.6. Time of Day and Optimal Response to BLT

Morning exposure to BLT may not have been necessary for a clinical response in this small group of depressed adolescents. Our findings are similar to reports in adults with SAD, which showed a 70% response rate (fifteen out of twenty-one with seasonal depression) from evening bright light [41]; in adults with bipolar major depression who utilized midday light as adjunctive therapy [14]; in adolescents with non-seasonal MDD who utilized afternoon LT (between 1600 and 2000 h) [32]; and in adolescents with SAD who reported a stable response to midday BLT but did not tolerate the activating effects of bright morning light [19]. In some patient groups including adolescents, the response to BLT may be independent of the time of day [41], but further studies with larger sample sizes are needed to systematically investigate this question.

### 3.7. Adverse Events

BLT in this study was well-tolerated, with no participants withdrawing due to adverse events. Somatic symptoms captured at baseline on systematic assessment either persisted at unchanged levels throughout the study or tended toward improvement. These findings are consistent with other BLT studies in adolescents in which adverse events were systematically assessed [17].

### 3.8. Actigraphy

For the first time, we found that BLT increased actigraphic-derived sleep efficiency and shortened sleep onset latency compared to DRL in our outpatient light treatment study on adolescents with depression. He et al. also found that exposure to morning bright light versus regular office light yielded a higher sleep efficiency, as well as a smaller fragmentation index and a shorter time in bed, in non-depressed college students [42]. By contrast, Richardson and Gradisar [35] did not find actigraphic sleep differences between the green and red bright light conditions in adolescents with delayed sleep–wake phase disorder, and Grandner et al. also failed to detect actigraphic sleep differences between the 10,000-lux short wavelength and dim red light conditions in depressed college students [43]. Differences in results may be due to the type of light treatment modality (glasses or masks vs. light boxes), or the timing and/or duration of light treatment, among other factors.

### 3.9. Strengths/Limitations

In the study, the clinician rater was kept blind from the timing or dosing of light therapy, to reduce the likelihood that expectations could affect the scoring of clinical severity. The research team concealed the study hypotheses from participants and their families to avoid raising expectations and incurring biased self-reported ratings. Given the putative adverse effects from antidepressant chronotherapeutics, we systematically monitored for emergent suicide risk and mixed/hypomanic symptoms with repeated assessments to ensure that participants remained safe and to identify any participant with cycling mood symptoms. In this report, we present novel and promising findings of the safety, acceptability, and clinical response to BLT in outpatient youths with major depression. However, the small sample size, low diversity arising from a single recruitment site, lack of a parallel placebo control group and lack of an objective adherence monitor in the light boxes are factors that limit the generalization of these results. The blinded clinician’s knowledge of the sequence of the treatment protocol is a potential limitation. Knowing this, we utilized mainly self-report outcome measures to assess adolescent depression, anxiety, safety, and side effects. We relied on clinician ratings to evaluate the participants’ functioning and treatment-emergent hypomania. To further protect the “blind”, the timing of BLT was kept concealed from the blinded rater.

To our knowledge, this is the first study of bright light treatment with a placebo lead-in phase for moderate-to-severely depressed adolescents in an outpatient setting. We found bright light therapy to be feasible, tolerable, and acceptable to adolescent outpatients when we allowed them to adapt the treatment protocol to their lives and schedules. Our participants were recruited from primary care pediatrics, a setting where many adolescents first present with depression symptoms and from which referral to specialty care may be limited. Bright light therapy, if effective, may be an appealing treatment option that can be prescribed and monitored in a primary care setting. A randomized, parallel, placebo-controlled trial is imperative.

## 4. Methods

We performed this outpatient study at Ann & Robert H. Lurie Children’s Hospital of Chicago, in Chicago, IL. The Institutional Review Board of Lurie Children’s Hospital approved the study protocol. We obtained informed consent from all study participants involved in the study and their parents. Participants were recruited through a large community pediatric practice. Primary care pediatricians referred potential participants with depression and elevated scores on youth screening measures, Patient Health Questionnaire-9 (PHQ-9; [44]) (≥9) or PHQ-2 (≥3), without endorsed suicidality. The study was registered with ClinicalTrials.gov (National Clinical Trial Number: NCT05823090).

### 4.1. Stakeholder Engagement and Focus Group Interviews

There is little information regarding the feasibility and acceptability of BLT among adolescent outpatients. Prior to completing the study design, we brought together a focus group of adolescents who had a history of treatment for depression using a stakeholder academic resource panel (ShARP) sponsored by the Center for Community Health at Northwestern University. Five adolescents aged 14–18 years with diagnosed mood disorders participated in the 90 min ShARP panel. The participants had minimal prior knowledge of BLT and were not recruited as study participants. Three of the five ShARP participants indicated that they thought BLT could be helpful for youth with depression (two participants were “unsure”). Primary barriers identified by the youth included the time of day and duration that BLT would need to be used and the appearance of the device. The ShARP panel outcomes shaped the education provided to youth and families about the BLT intervention as related to acceptable activities while engaging in BLT and the use of behavioral nudges to prompt study reporting.

### 4.2. Inclusion Criteria

We included participants aged 12 to 18 years at the time of consent who had an English-speaking primary caregiver legally able to provide consent and who could contribute weekly mood ratings (in our study, all caregivers were parents). Participants were accepted for referral to the study by their primary care pediatrician with a Patient Health Questionnaire-9 (PHQ-9) (≥9) or PHQ-2 (≥3). Participants with major depressive disorder, as evaluated by a clinician (psychologist or psychiatrist) via structured interview, and with impairing symptoms, identified with a score of 4 or greater on the Clinical Global Impression—Severity (CGI-S) [45], were included. Participants with certain comorbid conditions such as cannabis use were included as long as the primary impairing disorder was depression. Exclusion criteria included current or past diagnoses of bipolar disorder, moderate-to-severe autism, schizophrenia, schizoaffective disorder, or intellectual disability; a major medical illness or ocular condition (e.g. glaucoma, retinal disease, macular degeneration) that would interfere with participation in the study; significant and imminent risk to self or to others; concurrent or recent (<4 months) antidepressant medication or new (<3 months) psychotherapy treatment for depression; or current use of melatonin, beta blockers, chloroquine, regular non-steroidal anti-inflammatory agents, or St. John’s Wort.

### 4.3. Diagnostic Interview and Clinical Ratings

The Mini-International Neuropsychiatric Interview for Children and Adolescents (MINI-KID) was used to confirm the clinical diagnosis according to the Diagnostic and Statistical Manual of Mental Disorders, Fifth Edition (DSM-5). The MINI-KID is a brief, DSM-5-keyed structured diagnostic interview for 6–17-year-old youth [46]. The MINI-KID assesses major psychiatric illnesses, using computerized, self-administered versions for youth and proxy interviews for caregivers. Responses to the MINI-KID are binary (yes/no), indicating the presence or absence of the disorder. Psychometric properties are comparable to other psychiatric interviews [47,48].

#### 4.3.1. Short Mood and Feelings Questionnaire (SMFQ)

The Short MFQ (SMFQ) is a 13-item version of the Mood and Feelings Questionnaire used to assess depressive symptoms in youth [49]. Youth and parent proxy measures are calculated as a total score (range = 0–26). A cutoff total score of 11 is recommended for the presence of a depressive disorder [30,34,50]. The SMFQ (13 items) has strong internal consistency (α = 0.92) [50].

#### 4.3.2. Clinical Global Impression—Severity and Improvement Scales (CGI-S and -I) 

The CGI-S is an objective clinician-rated score of illness severity ranging from 1 (not at all ill) to 7 (extremely ill) [45]. The CGI-I is an objective clinician rating of clinical improvement ranging from 1 (very much improved) to 7 (very much worse). CGIs have been commonly used in clinical trials [4].

#### 4.3.3. Screen for Child Anxiety Related Emotional Disorders (SCARED)

The SCARED is a 41-item questionnaire designed to assess a variety of anxiety symptoms, with parallel caregiver and youth versions [51]. The SCARED allows for the calculation of a total anxiety score (range = 0–82) and has a five-dimension structure, with subscale scores for separation anxiety, generalized anxiety, social anxiety, panic/somatic symptoms, and school avoidance. A total SCARED cutoff score of 25 is generally agreed upon as an indicator of the presence of an anxiety disorder [52,53]. The SCARED has demonstrated discriminant validity between anxious and non-anxious youth, strong internal consistency (coefficient α of approximately 0.90), and favorable psychometrics in treatment-seeking samples [53,54].

#### 4.3.4. Seasonal Pattern Assessment Questionnaire (SPAQ)

The SPAQ is a brief, self-administered screening tool utilized to assess for seasonal affective disorder (SAD) and explore changes in mood and behaviors across seasons. The global seasonality score (range = 0–24) is calculated from the sum of scores from six items that assess for seasonal changes in energy level, mood, sleep length, social activity, weight, and appetite. Participants indicate the degree to which they feel the item changes with the seasons (none = 0, a little = 1, sort of = 2, pretty much = 3, a lot = 4). The cutoff seasonality score of 11 or higher in our study was defined based on published data [55].

### 4.4. Assessments

Participants and their parent(s) consented for the study and completed a baseline assessment. The study team clinicians (R.B. and J.T.P.), who are both trained experts in the diagnosis and clinical management of child and adolescent mental disorders, reviewed the MINI-KID assessments, provided a confirmation of diagnosis, and assigned a severity rating according to the Clinical Global Impression—Severity (CGI-S). The adolescents completed the baseline SMFQ, SCARED, Columbia Suicide Severity Rating Scale, SPAQ, Systematic Assessment for Treatment Emergent Effects (SAFTEE) [56], Medical Outcomes Study Sleep Scale [57], and Morningness–Eveningness Questionnaire (MEQ) [58]. Parents completed the MINI-KID, SMFQ, Brief Child Mania Rating Scale (CMRS) [59], and Screen for Child Anxiety Related Disorders (SCARED) on their child. We assessed expectations of treatment at baseline and the final visit. The brief question on treatment expectancy was: “How much do you expect that doing light therapy affects (or will affect) your depression or mood problems? I expect my mood or depression problems will be: 1 = very much improved, 2 = much improved, 3 = minimally improved, 4 = unchanged, 5 = minimally worse, 6 = much worse, 7 = very much worse.” A schedule of the assessments used throughout the study is shown in Supplemental Table S1.

### 4.5. Light Therapy Protocol with Placebo Lead-In

Eligible participants were provided with a light box and an actigraphy wristwatch and provided standard instructions on the use of each. The active light box (Carex DayLight Classic Model) was a white fluorescent 4000-Kelvin unit that emits 10,000 lux and measures 33 cm × 40 cm. The placebo box emits 50 lux dim red light (DRL) and looks identical to the active unit. We selected DRL because the illumination is a plausible placebo in clinical trials of BLT for depressive disorders [12,14,60,61] and produces negligible effects on circadian rhythms [62] and mood responses [12,14,60,61]. Participants were instructed to position themselves 30–36 cm from the box with their faces fully exposed to the light. The actigraphy device was a Philips Actiwatch Spectrum Plus watch that collects activity level and sleep–wake information along with multiple light measurements and wearer adherence measures (described below). The light therapy dosing protocol was planned as follows: Weeks 0–2, 50 lux DRL × 30 min/day; weeks 3–4, 10,000 lux × 15 min/day; weeks 5–6, 10,000 lux × 30 min/day; weeks 6–8, 10,000 lux × 45 min/day. At week 2, the study coordinator (K.M.J.) dispensed the 10,000 lux BLT unit and the actigraphy watch (and picked up the DRL box). Participants and their parent met with 2 study clinicians at the end of each 2-week period. One “blinded” study clinician (R.B. or J.T.P.), who was kept concealed from the participant’s light dosing (# min/d and the time of day), conducted a brief follow-up interview every two weeks to assess participant mood ratings, safety, and functioning and assigned a clinical rating of severity (CGI-I and -S). The “non-blind” study clinician (D.K.S.) met with the participant at the same visit to review safety, side effects, and overall response and made recommendations based on patient and parent feedback. The clinician systematically recorded the dosing and timing of light box use (light exposure) in the dosing assessment form, based on level of improvement and any barriers to use. Even though some patients did not receive the above standard dosing schedule, the protocol was used as a novel tool to identify acceptable strategies to promote maximal adherence through evidence-based knowledge [25]. After the 8-week active study period, participants who experienced symptom remission were given the option to keep the light box and continue use. A treatment summary and ongoing treatment recommendations were provided to all participants’ primary care clinicians. Participants and parents were compensated for their time for completing study measures.

We addressed proper “masking” of the placebo nature of DRL in several ways. First, participants and parents/guardians/caregivers were kept blind to the aims and hypotheses. To accomplish this (and to ensure transparency), participants and their family were informed that at a certain time during the study, the light box would be adjusted to a unit that delivers a placebo light to help the study investigators understand how well the treatment light works in comparison. They were assured that the placebo unit would be given for only a part of the study (not for the entire duration) but not told when they would receive it. Participants and families were provided published information that as many as 30% (or one in three people) improved significantly with placebo light [12,14,60,61]. In the procedures, participants and families brought in the study light box for quality checks at weeks 2, 4, and 6. At the 2-week visit, the coordinator replaced the non-active unit with the unit configured with the active light. We did not assess for differences in expectation between DRL and BLT because doing so could unmask the placebo nature of DRL in a study of a visible intervention.

### 4.6. Adherence

The study coordinator made regular calls/texts every day to participants to inquire about their daily exposure times and to encourage proper adherence. To avoid forcing adolescents to wake earlier than preferred (which can produce detrimental effects on adolescent sleep and mood) to use the study light box, participants agreed to initiate BLT exposure in the morning at awakening in accordance with the dosing protocol. Given that the constraints imposed upon adolescents by schools (early morning start times by 0800 h), bus commutes (pick up by 0730 h), after school activities, and part-time jobs (landscaping, etc.) are considerable, the non-blind clinician systematically explored and documented whether morning use remained feasible and acceptable at follow-up visits. If not, the clinician made informed recommendations and recorded adjustments to the prescribed time of day [25,41,63] that were necessary to support the participants’ consistent use of the light therapy box, to optimize adherence and to strengthen the reliability of detecting response.

### 4.7. Safety Checks

The study participants completed weekly self-ratings of severity of depression, anxiety, suicidality, and side effects using the SMFQ, Columbia Suicide Severity Scale [64], SCARED, and SAFTEE. They received a daily text prompt from the study coordinator asking them to report the number of minutes they had used their light box. Parents rated the severity of depressive symptoms, anxiety, and hypomania using the SMFQ, SCARED and CMRS on their teen every two weeks during the study period.

### 4.8. Actigraphic Assessments

Actigraphy using a wrist actigraph with a light sensor (Actiwatch Spectrum Plus, Philips Respironics Healthcare, Bend, OR, USA) was used to measure a number of actigraphic-derived sleep, circadian, activity and light variables during the entire DRL and the entire BLT treatment phases. Actiwatches were worn on the non-dominant wrist and data were collected in 1 min intervals (using firmware version 01.01.0006, medium wake threshold) and processed using the Actiware software (version 6.1.0). Total activity counts per day (the sum of all physical activity counts for all epochs) and light metrics (white, red, green, and blue total exposure) were derived from the Actiware software. Days were excluded in which there were any missing nighttime sleep data or when the Actiwatch was not worn by the participant. In this preliminary study, we did not formally track whether the Actiwatch was covered by clothing. Actigraphic data were reviewed by two trained investigators (L.N.P. and N.G.) and were analyzed as in our prior studies [65,66,67,68,69].

### 4.9. Data Analysis

To visualize trends over time across all individuals, spaghetti plots were constructed for continuous outcomes with repeated measurements. The loess (local polynomial regression) smoothed line with degree 2 was included with a 95% confidence band in a gray shade. [https://rdrr.io/r/stats/loess.html; accessed on 1 November 2022]. The week 0, week 2 and week 8 differences were calculated and tested for statistical differences in R (https://www.R-project.org; accessed on 1 November 2022) using paired non-parametric methods due to the small sample size and the paired nature of the data. For continuous outcomes, Wilcoxon signed rank tests were used, and for binary outcomes, McNemar’s tests were used [70]. *p*-values were adjusted for multiple pairwise comparisons using the Holm–Bonferroni method [71]. To estimate treatment effect from the change in mood scores, we examined effect size using Cohen’s d [27]. Spearman correlation coefficients examined correlations between participant and parent SMFQ mood scores.

### 4.10. Statistical Analyses of Actigraphy Data

Wilcoxon signed rank tests (SPSS v26, SPSS Inc., Chicago, IL, USA) were used to compare means within each individual for differences in actigraphic-derived sleep, circadian, activity, and light measures in the entire DRL phase compared to the entire BLT phase in participants who wore the watch in both phases (n = 6). *p* < 0.05 was considered statistically significant, and Wilcoxon signed rank tests were two-tailed.

## Figures and Tables

**Table 1:** Change in child/parent depression mood scores (SMFQ) and clinician severity ratings (CGI-S) across the time points week 0, week 2, and week 8.

Measure	T1	T2	Median Change	V stat; p_adj	Effect Size (Cohen’s d)
SMFQ_child_score	week_0 (N = 9)	week_2 (N = 9)	7	42; 0.042 *	1.10
SMFQ_child_score	week_0 (N = 9)	week_8 (N = 8)	10	36; 0.042 *	3.52
SMFQ_child_score	week_2 (N = 9)	week_8 (N = 8)	4	32; 0.058	0.99
SMFQ_parent_score	week_0 (N = 9)	week_2 (N = 9)	5	39.5; 0.1	0.99
SMFQ_parent_score	week_0 (N = 9)	week_8 (N = 8)	7	28; 0.066	1.35
SMFQ_parent_score	week_2 (N = 9)	week_8 (N = 8)	1	13.5; 0.598	0.22
CGI-S_clinician	week_0 (N = 9)	week_2 (N = 9)	2	21; 0.067	1.12
CGI-S_clinician	week_0 (N = 9)	week_8 (N = 7)	2	28; 0.064	2.00
CGI-S_clinician	week_2 (N = 9)	week_8 (N = 7)	1	15; 0.067	1.73

* *p* < 0.05.

**Table 2:** Actigraphic sleep data during the dim red light (DRL) and bright light therapy (BLT) phases.

		DRL	BLT
		N = 6	N = 6
Sleep Variable	Total Sleep Time (min)	381.59 ± 43.66	388.06 ± 55.22
	Sleep Duration (min)	431.05 ± 42.55	437.77 ± 54.40
	Sleep Onset Latency (min)	14.23 ± 8.31	9.39 ± 5.60
	Sleep Onset (clock hour)	24.13 ± 1.33	24.51 ± 0.83
	Sleep Offset (clock hour)	7.32 ± 1.17	7.80 ± 1.15
	Sleep Midpoint (clock hour)	3.73 ± 1.20	4.16 ± 0.89
	Wake Time (%)	11.17 ± 1.85	11.10 ± 1.70
	Sleep Time (%)	88.83 ± 1.85	88.90 ± 1.70
	Wake After Sleep Onset (min)	47.60 ± 5.03	48.30 ± 5.87
	Sleep Efficiency (%)	83.84 ± 2.07	85.00 ± 2.48 *

Values are presented as mean ± SD. * Sleep efficiency was significantly higher in the BLT phase than the DRL phase as assessed by Wilcoxon signed rank test (z = −1.99, *p* = 0.046).

## Data Availability

The data presented in this study are deidentified and available upon request from the corresponding author.

## Supplementary Materials

The following supporting information can be downloaded at: https://www.mdpi.com/article/10.3390/clockssleep6010005/s1, Table S1: Schedule of Assessments.

## Abbreviations

BLT	bright light therapy
CGI-S and -I	Clinical Global Impression Scale Severity and Improvement Scales
CMRS	brief Child Mania Rating Scale
DRL	dim red light
DSM-5	Diagnostic and Statistical Manual of Mental Disorders, Fifth Edition
IQR	interquartile range
MEQ	Morningness–Eveningness Questionnaire
MDD	major depressive disorder
MINI-KID	Mini-International Neuropsychiatric Interview for Children and Adolescents
PHQ-9	Patient Health Questionnaire-9
SAD	seasonal affective disorder
SCARED	Screen for Child Anxiety Related Disorders
SAFTEE	Systematic Assessment for Treatment Emergent Effects
ShARP	stakeholder academic resource panel (ShARP)
SMFQ	Short Mood and Feelings Questionnaire
SPAQ	Seasonal Pattern Assessment Questionnaire
