# Supplementary material for: Bright Light Therapy for Major Depressive Disorder in Adolescent Outpatients: A Preliminary Study
Source: Clocks Sleep. 2024 Jan 30;6(1):56–71. doi: 10.3390/clockssleep6010005 (PMC10885037; doi:10.3390/clockssleep6010005)
Supplement: Supplementary file 1 [file clockssleep-06-00005-s001.zip › clockssleep-2774047-supplementary.pdf]

Table S1. Schedule of Assessments.

**Construct**	**Measure**	Completed by:	Baseline	Weekly	Every 2 weeks	End of study
**SCREENING**						
Depression	Patient Health Questionnaire-9 or -2	Y				
**ELIGIBILITY**						
Major Depression	MINI-KID	P/Y	X			
Seasonality	Seasonal Pattern Assessment Questionnaire (SPAQ)	Y	X			
**PRIMARY OUTCOMES**						
Depression	Short Mood and Feelings Questionnaire (SMFQ)	P/Y	X	X		
Suicide	Columbia Suicide Severity Scale (CSSS)	Y	X	X		
Severity	Clinical Global Impressions - Severity and Improvement (CGI-S, I)	C	X		X	
Mania	Brief Child Mania Rating Scale (CMRS)	P	X		X	
Side effects	Systematic Assessment for Treatment Emergent Effects (SAFTEE, modified, 26-items)	Y	X	X		
**COVARIATES**						
Sleep	Medical Outcomes Study Sleep Scale	Y	X		X	
Anxiety	Screen for Child Anxiety Related Disorders (SCARED)	P/Y	X			X
Chronotype	Morningness-Eveningness Questionnaire	Y	X			
Process	Expectations/perceived effectiveness of treatment	Y	X			X
Sleep, activity, light	Actigraphy data from watch	Device		continuous		

P = parent, Y = youth, C = clinician
